# Changes over time in characteristics, resource use and outcomes among ICU patients with COVID‐19—A nationwide, observational study in Denmark

**DOI:** 10.1111/aas.14113

**Published:** 2022-08-02

**Authors:** Nicolai Haase, Ronni Plovsing, Steffen Christensen, Lone M. Poulsen, Anne C. Brøchner, Bodil S. Rasmussen, Marie Helleberg, Jens U. S. Jensen, Lars P. K. Andersen, Hanna Siegel, Michael Ibsen, Vibeke L. Jørgensen, Robert Winding, Susanne Iversen, Henrik P. Pedersen, Jacob Madsen, Christoffer Sølling, Ricardo S. Garcia, Jens Michelsen, Thomas Mohr, George Michagin, Ulrick S. Espelund, Helle Bundgaard, Lynge Kirkegaard, Margit Smitt, David L. Buck, Niels‐Erik Ribergaard, Helle S. Pedersen, Birgitte V. Christensen, Lone P. Nielsen, Esben Clapp, Trine B. Jonassen, Sarah Weihe, Kirstine la Cour, Frederik M. Nielsen, Emilie K. Madsen, Trine N. Haberlandt, Nick Meier, Anders Perner

**Affiliations:** ^1^ Department of Intensive Care Rigshospitalet Copenhagen Denmark; ^2^ Department of Anaesthesiology and Intensive Care Hvidovre Hospital Copenhagen Denmark; ^3^ Department of Anaesthesiology and Intensive Care Århus University Hospital Århus Denmark; ^4^ Department of Anaesthesiology Zealand University Hospital Køge Denmark; ^5^ Department of Anaesthesiology and Intensive Care Kolding Hospital Kolding Denmark; ^6^ Department of Anaesthesiology and Intensive Care Ålborg University Hospital Denmark; ^7^ Department of Infectious Diseases Rigshospitalet Copenhagen Denmark; ^8^ Department of Respiratory Medicine Herlev‐Gentofte Hospital Copenhagen Denmark; ^9^ Department of Anaesthesiology and Intensive Care Bispebjerg Hospital Copenhagen Denmark; ^10^ Department of Anaesthesiology and Intensive Care Herlev‐Gentofte Hospital Copenhagen Denmark; ^11^ Department of Anaesthesiology and Intensive Care North Zealand Hospital Hillerød Denmark; ^12^ Department of Cardiothoracic Anaesthesiology Rigshospitalet Copenhagen Denmark; ^13^ Department of Anaesthesiology and Intensive Care Herning Hospital Herning Denmark; ^14^ Department of Anaesthesiology and Intensive Care Slagelse Hospital Slagelse Denmark; ^15^ Department of Anaesthesiology and Intensive Care Zealand University Hospital Roskilde Denmark; ^16^ Department of Anaesthesiology and Intensive Care Viborg Hospital Viborg Denmark; ^17^ Department of Anaesthesiology and Intensive Care Esbjerg Hospital Esbjerg Denmark; ^18^ Department of Anaesthesiology and Intensive Care Odense University Hospital Odense Denmark; ^19^ Department of Anaesthesiology and Intensive Care Svendborg Hospital Svendborg Denmark; ^20^ Department of Anaesthesiology and Intensive Care Horsens Hospital Horsens Denmark; ^21^ Department of Anaesthesiology and Intensive Care Randers Hospital Randers Denmark; ^22^ Department of Anaesthesiology and Intensive Care Åbenrå Hospital Åbenrå Denmark; ^23^ Department of Neuroanaesthesiology Rigshospitalet Copenhagen Denmark; ^24^ Department of Anaesthesiology and Intensive Care Holbæk Hospital Holbæk Denmark; ^25^ Department of Anaesthesiology and Intensive Care Hjørring Hospital Hjørring Denmark; ^26^ Department of Anaesthesiology and Intensive Care Nykøbing Falster Hospital Nykøbing Falster Denmark; ^27^ Department of Anaesthesiology and Intensive Care Glostrup Hospital Copenhagen Denmark; ^28^ Department of Anaesthesiology and Intensive Care Bornholms Hospital Rønne Denmark

**Keywords:** comorbidities, COVID‐19, intensive care, mortality, SARS‐CoV‐2

## Abstract

**Background:**

Characteristics and care of intensive care unit (ICU) patients with COVID‐19 may have changed during the pandemic, but longitudinal data assessing this are limited. We compared patients with COVID‐19 admitted to Danish ICUs in the first wave with those admitted later.

**Methods:**

Among all Danish ICU patients with COVID‐19, we compared demographics, chronic comorbidities, use of organ support, length of stay and vital status of those admitted 10 March to 19 May 2020 (first wave) versus 20 May 2020 to 30 June 2021. We analysed risk factors for death by adjusted logistic regression analysis.

**Results:**

Among all hospitalised patients with COVID‐19, a lower proportion was admitted to ICU after the first wave (13% vs. 8%). Among all 1374 ICU patients with COVID‐19, 326 were admitted during the first wave. There were no major differences in patient's characteristics or mortality between the two periods, but use of invasive mechanical ventilation (81% vs. 58% of patients), renal replacement therapy (26% vs. 13%) and ECMO (8% vs. 3%) and median length of stay in ICU (13 vs. 10 days) and in hospital (20 vs. 17 days) were all significantly lower after the first wave. Risk factors for death were higher age, larger burden of comorbidities (heart failure, pulmonary disease and kidney disease) and active cancer, but not admission during or after the first wave.

**Conclusions:**

After the first wave of COVID‐19 in Denmark, a lower proportion of hospitalised patients with COVID‐19 were admitted to ICU. Among ICU patients, use of organ support was lower and length of stay was reduced, but mortality rates remained at a relatively high level.

**Editorial Comment**

This study assessed the temporal changes in the care of patients with COVID‐19 requiring intensive care unit (ICU) care in Denmark. The findings showed that while a lower ratio of patients with documented infections required ICU and they required less organ support, ICU mortality remained unchanged. This might reflect the effects of vaccines on disease severity and improvement in floor management of hypoxic patients, but also underscores that COVID‐19 remains a serious threat to the health of many patients, particularly elderly patients with a high degree of comorbidity.

## 1 INTRODUCTION

Since March 2020, the severe acute respiratory syndrome coronavirus 2 (SARS‐CoV‐2) has caused a pandemic and resulted in many hospitalised patients with coronavirus disease 2019 (COVID‐19) and severe respiratory failure.

After the first wave of the epidemic in Denmark, we reported data from all Danish intensive care unit (ICU) patients with COVID‐19 and found high use of organ support (i.e., invasive mechanical ventilation, renal replacement therapy [RRT] and extracorporeal membrane oxygenation [ECMO]), considerable stay in ICU and in hospital, an overall mortality of 37%. Male gender, higher age and the number of comorbidities were associated with higher mortality.^1^ These characteristics and outcomes may have changed after the first wave because of improved hospital care of patients with hypoxemia in general ward, the use of medical interventions against severe COVID‐19, in particular dexamethasone, and introduction of new SARS‐CoV‐2 variants.^2^

There are reports on potential changes in characteristics, resource use and outcomes of ICU patients with COVID‐19 between the first wave and subsequent time periods, but most studies are from single centres or regions.^3^, ^4^ Furthermore, the interpretation of the few nationwide studies is limited by lack of data on patient characteristics and resource use.^5^, ^6^ In addition, the periodic heavy strain on many healthcare systems has likely influenced the selection, use of organ support and outcomes of ICU patients,^7^, ^8^, ^9^ which may hamper the interpretation of any changes in these variables over time. Population‐based data from less stressed healthcare systems may better reflect if any changes have occurred in characteristics, use of organ support and outcomes in ICU patients with COVID‐19 unrelated to those inflicted by variations in patient surges. As such, Danish ICUs may provide valid data, because the Danish healthcare system was never overwhelmed, and the triage criteria for admitting patients with COVID‐19 into Danish ICUs may have been relatively stable over the course of the pandemic.

We therefore compared the characteristics, use of resources and mortality in all Danish ICU patients with COVID‐19 admitted during and after the first wave of the epidemic.

## 2 METHODS

This study was a nationwide, retrospective observational study of all ICU patients with COVID‐19 in Denmark from 10 March 2020 to 30 June 2021. The data from patients during the first wave (10 March 2020 to 19 May 2020) were previously reported.^1^ In the present report, we compared the patients in the first wave with those subsequently admitted (20 May 2020 to 30 June 2021). The Danish Patient Safety Authority and Capital Region granted access to the patient files and waived consent from the individual patients due to the retrospective nature of the study according to Danish law (ref. no. 31‐1521‐293, R‐21004283). Ethics committee approval is not required for this type of study in Denmark. The database was designed in accordance with the European Union General Data Protection Regulation.

### 2.1 Setting

All 29 ICUs in the 27 hospitals in Denmark admitting patients with COVID‐19 contributed to the study.

### 2.2 Study population

We manually screened all ICU patients from the date of the first detected case with COVID‐19 (27 February 2020) in Denmark to 30 June 2021. Patients of all ages with a positive real‐time polymerase chain reaction (RT‐PCR) test for SARS‐CoV‐2 either before or during ICU admission were included. The database was closed on 1 September 2021, 3 months after the inclusion of the last patient. The total number of SARS‐CoV‐2 positive cases hospitalised patients with COVID‐19 in the two periods and vaccination rates were obtained from Statens Serum Institute (covid19.ssi.dk), which links the patients with SARS‐Cov‐2 positive airway samples with hospital admission and vaccination data from the five regions of Denmark using the unique civil registration number.

### 2.3 Data collection

Dedicated study personnel entered the following information retrieved from the patient files into a RedCap research database: (1) administrative data: admitting hospital, date of hospital and ICU admission, and ICU and hospital discharge; (2) demographics: age, gender, height, weight, time from onset of symptoms to hospital admission and to ICU admission; (3) chronic comorbidities: hypertension (use of antihypertensive medication), ischemic heart disease (previous myocardial infarction, coronary stenting, stable or unstable angina) or heart failure (left‐ventricular ejection fraction <40% or New York Heart Association Functional Classification [NYHA] 3 or 4), chronic pulmonary disease (use of inhalers), chronic kidney disease (estimated glomerular filtration rate < 60 ml/min/1.73 m^2^), diabetes (use of any antidiabetic drug [oral or injection]), active cancer, hematologic cancer (leukaemia, lymphoma or myeloma) and immunocompromise (congenital immunodeficiency, human immunodeficiency virus [HIV] or use of radiotherapy, chemotherapy or systemic prednisolone or other immunosuppressive agent within the last 6 months); (4) ICU use of invasive mechanical ventilation, RRT and ECMO; and (5) follow‐up data: ICU length of stay, hospital length of stay and mortality at 90 days after ICU admission.

### 2.4 Statistics

First, we described patient and admission characteristics using common descriptive statistics. 95% confidence intervals (CIs) of the proportion were calculated using the binomial proportion. Second, we compared characteristics, use of organ support and length of stay between the two periods using Chi‐square, Wilcoxon rank‐sum test and Cochran–Armitage test for trend as appropriate. Third, we analysed the risk of death according to burden of comorbidity, organ support, body mass index (BMI), age and admission during the first wave versus later using Chi‐square and Cochran–Armitage tests for trend as appropriate. Finally, we assessed baseline risk factors for mortality at 90 days using uni‐ and multivariate logistic regression in the entire cohort. All variables were included in the multivariate model including admission during the first wave versus later. Missing data were limited and no imputations were made. All statistical analyses were performed using SAS Enterprise Edition 3.8, SAS Institute Inc.

## 3 RESULTS

A total of 1374 patients with COVID‐19 were admitted to Danish ICUs from 10 March 2020 to 30 June 2021, among whom 326 were admitted until 19 May 2020 and 1048 after that date.

Among all hospitalised patients with COVID‐19 in Denmark, 2418 were admitted until 19 May 2020 and 13,266 from 20 May 2020 to 30 June 2021. Thus, 13% of the hospitalised patients with COVID‐19 were admitted to an ICU during the first wave as compared to 8% after the first wave.

**FIGURE 1:**
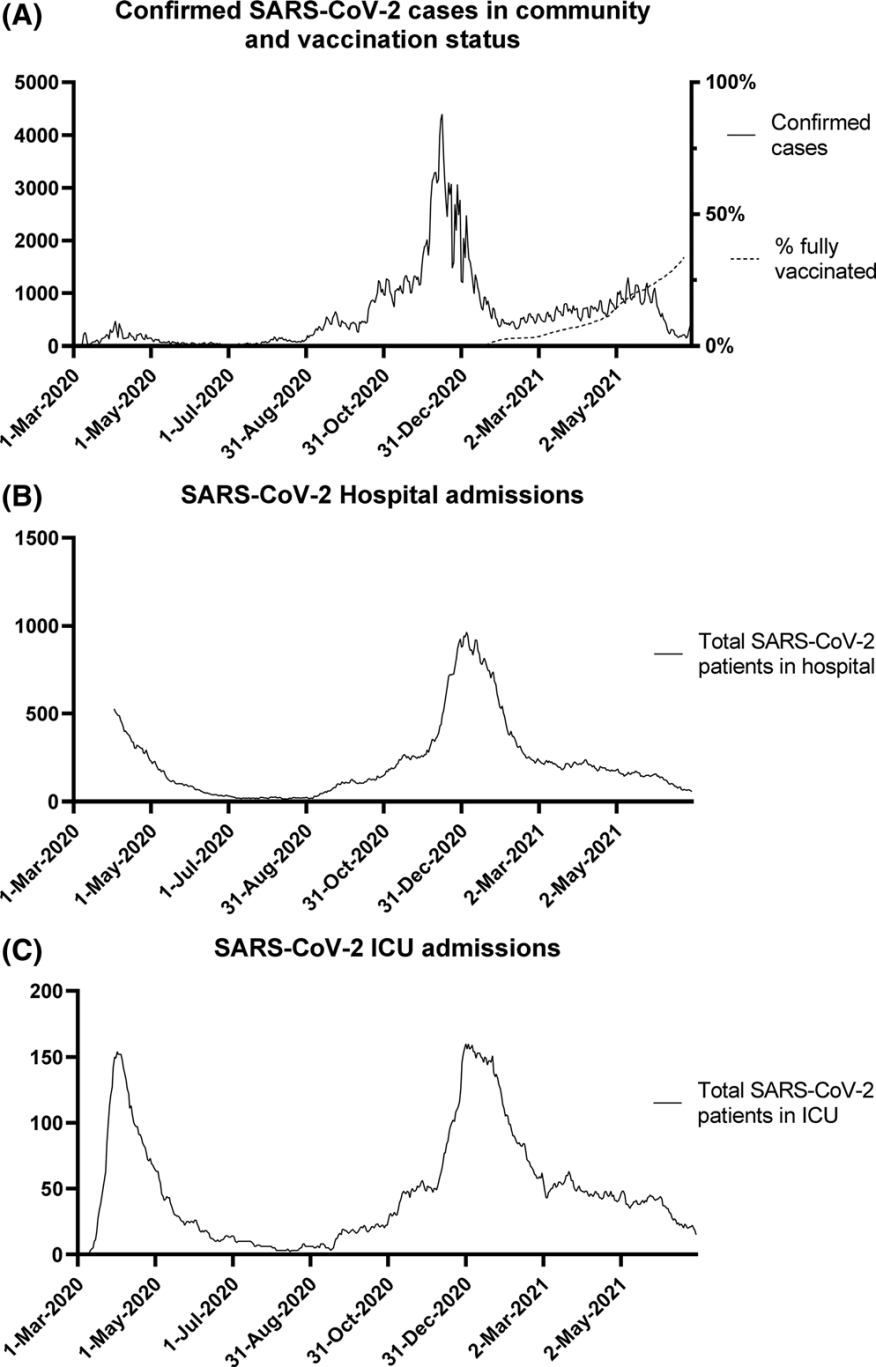
Confirmed SARS‐CoV‐2 cases (panel A), vaccination status (panel A), SARS‐CoV‐2 hospital admissions (panel B) and SARS‐CoV‐2 ICU admissions in Denmark from 1 March 2020 to 30 June 2021. In September 2020, an extensive public SARS‐CoV‐2 testing programme was launched to increase the number of confirmed cases. ICU, intensive care unit; SARS‐CoV‐2, severe acute respiratory syndrome coronavirus 2.

In Danish ICUs, the first COVID‐19 wave peaked in the last week of March 2020. From mid‐April 2020, the number of daily ICU admissions due to COVID‐19 declined to very low numbers from May to August 2020, after which the number of admissions increased again peaking end‐December 2020. From March to June 2021, the number of new admissions was at a lower and stable level. Timelines of SARS‐CoV‐2 positive cases, vaccination rates, hospital admissions and ICU admissions are shown in Figure 1.

Comorbidities, admission data and use of resources in ICU patients with COVID‐19 during and after the first wave.

There were no major differences in the age, gender, comorbidities, or time from onset of symptoms to admission between the ICU patients admitted with COVID‐19 during the first wave and those admitted after (Tables 1). During both periods, slightly less than one third had no known comorbidity.

**TABLE 1:** Demographics and comorbidities among all Danish ICU patients with COVID‐19 stratified by admission during or after the first epidemic wave

	All patients	Admitted during first wave (until 19 May 2020)	Admitted after the first wave (20 May 2020 to 30 June 2021)	*p* Values
Number of patients	1374	326	1048	–
Male gender, *N* (%)	930 (68)	239 (73)	691 (66)	.01
Age, years	67 (57–75)	69 (59–75)	66 (57–74)	.08
Body mass index	28 (25–33)	27 (24–31)	29 (25–33)	<.001
Time from initial symptom to hospital admission, days	7 (4–10)	7 (4–10)	7 (4–10)	.20
Time from hospital to ICU admission, days	1 (0–4)	2 (1–4)	1 (0–4)	.007
*Comorbidities, N (%)*				
Hypertension	732 (53)	164 (50)	568 (54)	.22
Ischemic heart disease	199 (15)	43 (13)	156 (15)	.45
Heart failure	66 (5)	15 (5)	51 (5)	.85
Chronic pulmonary disease	261 (19)	65 (20)	196 (19)	.62
Chronic kidney disease	199 (14)	40 (12)	159 (15)	.19
Liver cirrhosis	12 (1)	3 (1)	9 (1)	.92
Diabetes	324 (24)	67 (21)	257 (25)	.14
Active cancer	48 (3)	15 (5)	33 (3)	.21
Haematological malignancy	74 (5)	13 (4)	61 (6)	.20
Immunosuppressed	155 (11)	34 (10)	121 (12)	.58
None of the above	379 (28)	94 (29)	285 (27)	.56

*Note*: Continuous variables are medians and interquartile ranges. Body mass index was missing for 31 patients during the first wave and 112 after the first wave.

Abbreviation: ICU, intensive care unit.

The use of invasive mechanical ventilation, RRT and ECMO was reduced after the first wave and so was the length of stay both in ICU and in hospital (Table 2). The time on invasive mechanical ventilation was identical in the two time periods (Table 2).

**TABLE 2:** Organ supportive interventions, length of stay and mortality among all Danish ICU patients with COVID‐19 stratified by admission during or after the first wave of the epidemic

	All patients	Admitted during first wave (until 19 May 2020)	Admitted after first wave (20 May 2020 to 30 June 2021)	*p* Value for difference between time periods
Number of patients	1374	326	1048	
Use of organ support, *N* (%)				
Invasive mechanical ventilation	877 (64)	265 (81)	612 (58)	<.0001
Renal replacement therapy	224 (16)	84 (26)	140 (13)	<.0001
ECMO	57 (4)	25 (8)	32 (3)	.0003
Duration of organ support (days), median (IQR)				
Mechanical ventilation	13 (7–24)	13 (7–21)	13 (6–24)	.64
		min 1, max 69	min 1, max 240	
ICU length of stay, days				
All patients	11 (5–21)	13 (6–22)	10 (5–21)	.003
ICU survivors	9 (5–19)	13 (7–22)	8 (4–17)	<.0001
Hospital length of stay, days				
All patients	18 (10–30)	20 (11–32)	17 (10–29)	.039
Hospital survivors	19 (11–32)	24 (15–34)	17 (11–31)	<.0001
Mortality				
Died, *N* (% [95% CI])	493 (36% [33–38])	124 (38% [33–44])	369 (35% [32–38])	.39
In hospital	472	118	354	
After hospital discharge	21	6	15	
28‐day mortality, *N* (% [95% CI])	381 (28% [25–30])	93 (29% [24–34])	288 (27% [25–30])	.78
90‐day mortality, *N* (% [95% CI])	481 (35% [33–38])	118 (36% [31–42])	363 (35% [32–38])	.74

*Note*: 90‐day mortality data were missing for four non‐Danish patients after the first wave.

Abbreviations: CI, confidence interval; ECMO, extracorporeal membrane oxygenation; ICU, intensive care unit; IQR, interquartile range; OR, odds ratio.

### 3.1 Risk factors for death at 90 days among all ICU patients with COVID‐19

The mortality at 90 days was 481/1370 (35%, 95% CI 33%–38%) overall and identical in the two time periods (Table 2). In the unadjusted analyses, higher age and pre‐existing cardiovascular, pulmonary and kidney disease, diabetes, cancer, and being immunocompromised were all risk factors for 90‐day mortality, whereas gender and BMI were not (Table 3). In the adjusted analysis, higher age, pre‐existing heart failure, chronic kidney or pulmonary disease, and active cancer were risk factors for 90‐day mortality, whereas hypertension and diabetes were not. In neither the unadjusted nor the adjusted analyses, admission during or after the first wave was associated with mortality (Table 3).

**TABLE 3:** Logistic regression of risk factors for death at 90 days among all Danish ICU patients with COVID‐19

	Univariate analysis		Multivariate analysis	
Risk factor	OR (95% CI)	*p* Value	OR (95% CI)	*p* Value
Male gender (ref. female)	1.14 (0.90–1.45)	.28	1.20 (0.89–1.61)	.23
Admitted after versus during first wave (ref. first wave)	0.94 (0.73–1.22)	.64	1.04 (0.77–1.42	.79
Ischemic heart disease	1.74 (1.29–2.37)	.0003	0.79 (0.54–1.18)	.25
Heart failure	5.36 (3.08–9.32)	<.0001	3.25 (1.72–6.16)	.0003
Hypertension	1.79 (1.42–2.24)	<.0001	0.97 (0.72–1.30)	.84
Chronic pulmonary disease	1.66 (1.26–2.19)	.0003	1.60 (1.15–2.21)	.005
Chronic kidney disease	2.87 (2.11–3.89)		2.10 (1.46–3.01)	<.0001
Liver cirrhosis	1.32 (0.42–4.19)	.63	0.72 (0.15–3.42)	.68
Diabetes	1.47 (1.14–1.90)	.003	1.20 (0.88–1.63	.26
Active cancer	3.53 (1.93–6.45)	<.0001	2.26 (1.16–4.41)	.02
Haematologic cancer	1.91 (1.20–3.07)	.007	1.41 (0.79–2.53)	.25
Immunocompromised	1.92 (1.37–2.69)	<.0001	1.39 (0.90–2.14)	.14
*Age*				
<50	0.26 (0.15–0.44)	<.0001	0.33 (0.18–0.59)	<.0001
50–59	0.47 (0.32–0.70)	<.0001	0.47 (0.30–0.73)	<.0001
60–69	1	–	1	–
70–79	2.17 (1.62–2.91)	<.0001	1.84 (1.33–2.54)	<.0001
80+	4.68 (3.04–7.21)	<.0001	4.22 (2.62–6.80)	<.0001
*Body mass index*				
<18	1.02 (0.34–3.08)	.91	0.77 (0.22–2.74)	.58
18–24.9	1.25 (0.92–1.70)	.08	1.09 (0.77–1.53)	.78
25–29.9	1	–	–	–
30–34.9	0.78 (0.56–1.07)	.17	0.83 (0.58–1.19)	.21
35–39.9	0.80 (0.51–1.25)	.35	1.10 (0.67–1.82)	.78
>40	1.00 (0.63–1.59)	.84	1.60 (0.93–2.74)	.07

*Note*: Body mass index was missing for 31 patients during the first wave and 112 after the first wave. 90‐day mortality data were missing for 4 non‐Danish patients after the first wave. Only complete cases were analysed.

Abbreviations: CI, confidence interval; ICU, intensive care unit; OR, odds ratio.

**FIGURE 2:**
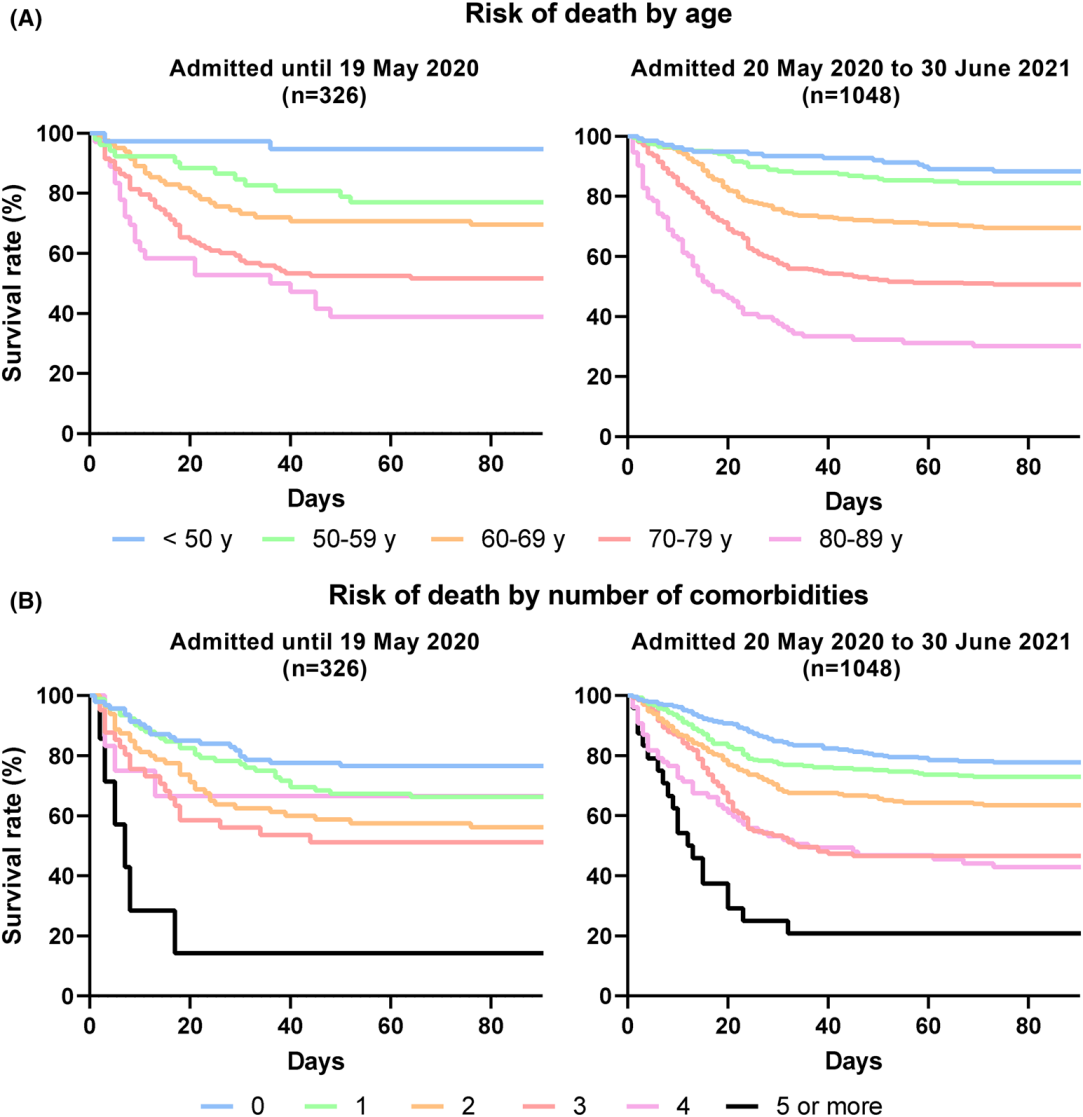
Mortality in Danish ICU patients with COVID‐19 stratified by age (panel A) and the number of chronic comorbidities (panel B) during and after the first epidemic wave. ICU, intensive care unit.

Mortality increased with increasing age and increasing number of comorbidities (Table 3 and Figure 2), trends that appeared to be similar during and after the first wave.

## 4 DISCUSSION

In this cohort study of all 1367 Danish patients with severe COVID‐19 admitted to ICU until 1 July 2021, a markedly smaller proportion among all hospitalised patients was admitted to ICU after the first wave of the epidemic. Among those admitted to ICU, fewer patients received invasive ventilation and markedly fewer received RRT and ECMO after the first wave. ICU patients admitted after the first wave also spent less time both in ICU and in hospital. Among the ICU patients, the mortalities at Days 28 and 90 were 28% and 35%, respectively, and similar during and after the first wave. The risk factors for fatal outcome were higher age and the burden of comorbidity, including heart failure, chronic pulmonary and kidney disease and active cancer. We did not find significant associations between gender, higher BMI, hypertension, ischaemic heart disease or diabetes and death in the multivariate analysis.

The number of SARS‐CoV‐2 positive cases in society, hospital admission rates and ICU admissions rates all depend on several factors including the dominating SARS‐CoV‐2 variant, time of year, society‐level and individual‐level preventive measures as well as vaccination rates. We do not hold data on the SARS‐CoV‐2 variants in our cohort, but the wild‐type variant was dominated until March 2021, where it was replaced by the alpha (B.1.1.7, UK) variant. In April 2021, the delta variant (Indian) was detected for the first time in Denmark, but it did not become dominant during the study period.

Society‐level preventive measures were adjusted according to case numbers and disease burden in hospitals several times during the study period, which obviously had an impact on ICU‐admission rates. The effect of specific preventive measures on ICU‐burden cannot be assessed in the present study.

The introduction of vaccines from early 2021 seems to have lowered both infection rates and admission rates to hospitals and ICUs. Those at risk of severe disease were prioritised in the vaccination programme, which relied mainly on Comirnaty (Pfizer/BioNTech). Again, this is difficult to further analyse in detail, but full vaccine coverage in Denmark was not yet established during the study period.

The reasons for the differences observed during and after the first epidemic wave may have been influenced by some of the above factors, but cannot be assessed in the present study. Patient's characteristics were similar between the two time periods despite a markedly lower proportion of hospitalised patients being admitted to ICU after the first wave. This suggests that there were no major differences in the ICU admission criteria during and after the first wave, but as we did not collect disease severity at time of admission this may not have been captured. Better care of hypoxemic patients on the hospital wards, including the use of dexamethasone,^2^ as well as the wide implementation of high flow nasal oxygen on general wards could be likely explanations for the reduced proportion of patients progressing to the need of intensive care. However, this cannot be further substantiated because there are limited Danish data available on the characteristics of hospitalised COVID‐19 patients outside the ICU. In line with our data, the Intensive Care National Audit and Research Center in the United Kingdom reported that 10%–15% of hospitalised patients were admitted to ICU during the first wave after which the fraction was reduced to 6%–10%.^10^The management in the ICU changed after the first wave with less use of invasive mechanical ventilation, RRT and ECMO. This may represent a change in the indications for the use of these invasive techniques in Danish ICUs. Similar trends have been observed in other hospitals, regions and countries.^3^, ^4^, ^5^, ^6^, ^11^

As for the use of invasive mechanical ventilation, there were likely early concerns about the safety of patients and staff of the use of open systems for oxygen supplementation.

Despite lower use of organ supportive interventions in Danish ICUs after the first wave, mortality remained relatively high and similar between the two time periods. Again, the reasons for this cannot be assessed, but time period was not a risk factor for death in the analysis of all patients after the adjustment for significant risk factors. The relative high mortality may partly be attributed to the high median age of our ICU population, but taken together, it appeared that the disease progression in patients with COVID‐19 resulting in ICU admission was associated with considerable risk of death despite the reduction in use of organ support after the first wave.

The significant risk factors for death observed among all Danish ICU patients with COVID‐19 were for most parts as those observed in other cohort studies.^12^, ^13^, ^14^, ^15^ However, higher BMI was not associated with increased mortality in our cohort which is in contrast to other cohorts of critically ill patients with COVID‐19.^13^, ^16^ Our multivariate analysis did suggest that severe obesity (BMI above 40) was associated with increased mortality, but this was not statistically significant.

The main strength of our study was the complete nationwide cohort of COVID‐19 ICU patients. We were able to follow all patients throughout their hospital admission apart from four foreign patients who were transferred abroad before Day 90. Thus, we present high‐quality data collected manually from patient files, which were electronic in all hospitals.

The limitations include the relatively few numbers of patients admitted during the first wave, which reduced the power. Also, a limited number of clinical variables were included to increase the feasibility of data retrieval. Therefore, some important risk factors for mortality were not included, for example, pre‐admission frailty, markers of acute disease severity at ICU admission and end‐of‐life decisions in the ICU. The inclusion of variables in the multivariate analysis was data driven, which increases the risk of chance findings.

In conclusion, after the first epidemic wave of COVID‐19 in Denmark, a lower proportion of hospitalised patients was admitted to ICU. Among those patients, use of organ support was lower and length of stay reduced, but mortality rates remained at a relatively high level. Age, pre‐existing comorbidity and active cancer were associated with increased risk of death.

## AUTHOR CONTRIBUTIONS

Nicolai Haase, Ronni Plovsing, Lone Musaeus Poulsen, Steffen Christensen, Anne Craveiro Brøchner, Bodil Steen Rasmussen, Marie Helleberg, Jens Ulrik Stæhr Jensen and Anders Perner contributed to the study conception and design. All authors contributed substantially to data acquisition. Data analyses were performed by Nicolai Haase. The first draft of the manuscript was written by Anders Perner and all authors commented on previous versions of the manuscript. All authors read and approved the final manuscript.

## Data Availability

Anonymised raw data may be available upon request from the corresponding author if certain legal requirements are fulfilled by the receiving party. SAS code available upon request from the corresponding author.
